# Comprehensive Analysis of Cold-Cracking Ratio for Flux-Cored Arc Steel Welds Using Y- and y-Grooves

**DOI:** 10.3390/ma14185349

**Published:** 2021-09-16

**Authors:** Hyunbin Nam, Jaeseok Yoo, Kwanghee Yun, Guo Xian, Hanji Park, Namkyu Kim, Sangwoo Song, Namhyun Kang

**Affiliations:** 1 Department of Joining Technology, Korea Institute of Materials Science, Changwon 51508, Korea; hbnam12@KIMS.re.kr (H.N.); swsong@kims.re.kr (S.S.); 2 Department of Welding Engineering R&D, Daewoo Shipbuilding & Marine Engineering Co., Ltd., Geoje-si 53302, Korea; yjs0303@dsme.co.kr (J.Y.); gjan@dsme.co.kr (K.Y.); 3 Department of Materials Science and Engineering, Pusan National University, Busan 46241, Korea; guoxian1989@gmail.com (G.X.); hjpark89@pusan.ac.kr (H.P.); 4 Department of Authorized Nuclear Inspection, Korea Institute of Materials Science, Changwon 51508, Korea; nkkim@kims.re.kr

**Keywords:** cold crack, flux-cored arc welding, welding materials, diffusible hydrogen, groove shape, stress concentration, microstructure, hardness

## Abstract

This study investigates various factors that influence the cold-cracking ratio (CCR) of flux-cored arc welds through Y- and y-groove tests. Factors affecting the CCR include the alloy component, diffusible hydrogen content, microstructure, hardness, and groove shape. In weld metals (WMs; WM375-R and WM375-B) of a low-strength grade, the diffusible hydrogen content has a more significant effect on the CCR than the carbon equivalent (*C_eq_*) and microstructure. However, the combined effects of the microstructure and diffusible hydrogen content on the CCR are important in high-strength-grade WM. The CCR of the WM increased upon increasing *C_eq_* and the strength grade because hard martensite and bainite microstructures were formed. Moreover, y-groove testing of the 500 MPa grade WM revealed a more significant CCR than that of the 375 MPa grade WM. Therefore, in high-strength-grade WMs, it is necessary to select the groove shape based on the morphology in the real welds.

## 1. Introduction

Energy resources in polar regions and abysses are receiving increasing attention owing to the greater possibility of economical production of crude oil and natural gas resources. Because the offshore plants and vessels in such areas are operated in extreme environments, high-strength structural steel with adequate low-temperature toughness is required [1,2]. Alloy elements are normally employed to ensure these properties. However, the weldability of alloyed steel is normally degraded because of the increase in the carbon equivalent (*C_eq_*) of the welds [3,4]. Cold cracking generally occurs in the heat-affected zone (HAZ) of normal carbon steel after welding at a temperature lower than 200 °C. Furthermore, high-strength steel has been reported to exhibit cold cracking in the weld metal (WM) rather than in the HAZ [5,6].

Cold cracking is known to be caused by complex interactions among factors such as the amount of diffusible hydrogen, low-temperature transformed microstructure, and residual tensile stress [7,8,9,10]. As the application of low-hydrogen-type welding flux has recently increased, the contribution of diffusible hydrogen to cold cracking has decreased correspondingly. Hence, under constant residual stress, the microstructure is the primary factor affecting cold cracking [11]. Previous studies on the microstructure of WMs have focused on imparting the WMs with high strength and toughness. Most studies have investigated the correlation between the mechanical properties and microstructural fraction as a function of the chemical composition [12,13,14]. With an appropriate fraction of acicular ferrite, it is possible to obtain WMs with high strength and toughness [15,16,17,18]. However, martensite is the hardest phase of the steel microstructure, and cold cracking frequently occurs in the martensite microstructure. This is because martensite easily traps diffusible hydrogen and undergoes a volume expansion during the phase transformation; thus, it is subjected to a residual tensile stress, which reduces the cold-cracking resistance [19,20]. 

To extend the applicability of high-strength steel, WMs requiring no or low preheating temperatures and exhibiting a reduced cold-cracking ratio (CCR) are being developed. The ISO17642-2 standard stipulates y-groove testing for CCR [21]. The inclusion larger than 2 μm was reported to increase the crack nucleation probability of high-strength steel welds [13]. Several studies have also been conducted based on Y-groove testing [13,22,23]. However, few studies provide a detailed analysis of the effect of diffusible hydrogen and groove shape on the CCR for steel with various grades of strength. Therefore, this study was conducted to comprehensively investigate the effects of these factors on CCR for steel with yield strengths of 235 and 500 MPa.

## 2. Materials and Experimental Procedures

The base metals (BMs) used in the Y/y-groove tests were YS 235 and 500 MPa grade steel plates with a thickness of 40 mm. Table 1 lists the chemical compositions of the BMs and WMs. Various WMs with strength levels of 375 and 500 MPa were used for flux-cored arc (FCA) welding. The types of flux adopted in this study are denoted as follows: R for rutile flux and B for basic flux; the types of sheath employed are represented as follows: F for folded type and S for seamless type. The diffusible hydrogen content of the WM was measured using the hot extraction method, with reference to ISO 3690 [24]. The carbon equivalent (*C_eq_*) and weld cracking parameter (*P_cm_*) of the WM were calculated using the following equations:

(1) $$C_{eq}\left(IIW\right)=C+Mn/6+\left(\left(Cr+Mo+V\right)\right)/5+\left(\left(Ni+Cu\right)\right)/15\;$$

(2) $$P_{cm}=C+Si/30+Mn/20+Cu/20+Ni/60+Cr/20+Mo/15+V/10+5B$$

The FCA welds with flux type B and sheath type S exhibited lower diffusible hydrogen contents than the welds with flux type R and sheath type F welds. The high-strength WMs (500 MPa) had larger values of *C_eq_* and *P_cm_* than the low-strength WMs (375 MPa).

**Figure 1:**
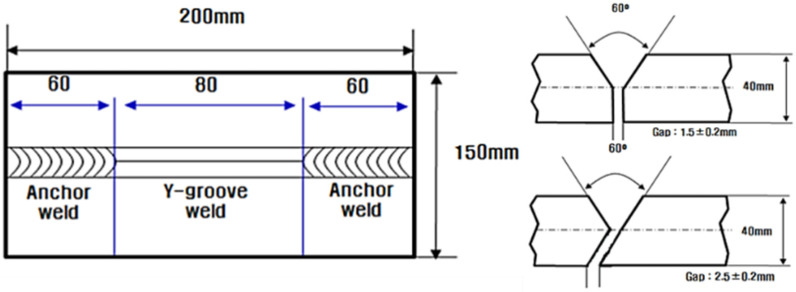
Schematics of Y- and y-groove tests.

The cold-cracking evaluation was performed following the ISO17642-2 standard [21], which stipulates y-groove testing. Furthermore, the cold-cracking resistance of the WM was evaluated based on the Y-groove test and the results were compared with those of the y-groove test. Figure 1 shows schematic diagrams of the Y- and y-groove tests.

The welding voltage and current for the fabrication of the Y/y-groove specimens were 25 V and 220 A, respectively, with no preheating (at 20 °C). The welding speed was 42 cm/min, and the welding heat input was 8 kJ/cm. After welding, the occurrence of cold cracking was determined by cutting the weld zone into five equal parts after 48 h at 20 °C. A macro-observation of the five cross sections was performed using optical stereoscopic microscopy. The CCR was calculated using the following equation:

(3) Cs=Hc/H×100 

where *Cs* is the CCR in a cross section (%), *Hc* is the root cracking height (mm), and *H* is the minimum thickness of the test beads (mm).

The Y- and y-groove welds were etched with a 2% Nital solution, and the microstructures were analyzed using light optical microscopy (LOM) and scanning electron microscopy (SEM). The microstructural type was defined based on the classification provided by the International Institute of Welding (IIW), as follows: acicular ferrite (AF), grain-boundary ferrite (GBF), Widmänstatten ferrite (WF), bainite (B), martensite (M), and martensite–austenite (MA) phases. The microstructural fractions of AF, GBF, and WF were analyzed using LOM photographs measured at 500× magnification, based on a point-counting method. For the B, M, and MA phases, a quantitative analysis using SEM was conducted because of the fine grain size of the microstructure. The hardness of the WM was measured using a Vickers microhardness tester. A load of 0.5 kg was applied for 10 s, and a position 0.5 mm under the surface of the test piece was measured at 0.5 mm intervals.

To simulate the welding process, the finite-element multi-physics software SYSWELD (ESI) was used to construct a thermal model. The governing differential equation for the transient thermal analysis was:

(4) $$\rho c_{p}\;\left(\partial T/\partial t\right)=\;\partial /\partial x\;\left[k_{x}\;\left(\partial T/\partial x\right)]+\partial /\partial y\;[k_{y}\;\left(\partial T/\partial y\right)]+\partial /\partial z\;[k_{z}\;\left(\partial T/\partial z\right)\right]+\dot{q}$$

where ρ is the density of the conducting medium; $c_{p}$ is the specific heat of the medium; $k_{x}$, $k_{y}$, and $k_{z}$ are the thermal conductivities of the medium in the *x*-, *y*-, and *z*-directions, respectively; t is time; and $\dot{q}$ is the total heat input based on Goldak’s double-ellipsoidal model [25]. The parameters used in the study are listed in Table 2. In this table, the notations a_f_ and a_r_ represent the front and rear lengths of the double-ellipsoidal heat source, respectively. In addition, b and c are the width and depth of the double ellipsoid, respectively, which define the size and shape of the ellipses. In addition, the heat transfer coefficient was 5.7 W/m^2^K, the thermal conductivity was 48.7 W/Mk, and the density was 7.78 kg/m^3^. To simplify the finite-element thermal analysis, the following assumptions were made:

1. The initial temperature of the model is 20 °C.
2. The fluid flow in the molten pool is negligible.

The heat transfer from the model to the environment is defined at the surface of the model as convective heat dissipation and radiation into the environment at 20 °C.

## 3. Results and Discussion

### 3.1. Effect of Alloying Constituent and Diffusible Hydrogen Content on Cold Cracking for Y-Groove Test

**Figure 2:**
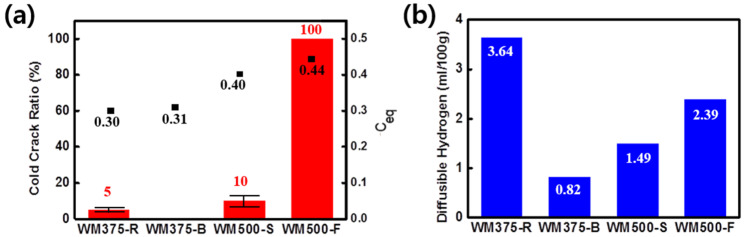
Effect of (**a**) *C_eq_* and (**b**) diffusible hydrogen content on CCR for various WMs.

Figure 2a,b present the CCRs of the WMs as a function of the alloying component and diffusible hydrogen content, respectively. The CCRs of WM375-R and WM375-B were 5% and 0%, respectively, for the YS 375 MPa-grade FCA welds (*C_eq_* = 0.3–0.31). WM500-S and WM500-F exhibited CCRs of 10% and 100%, respectively, for the YS 500 MPa-grade FCA welds (*C_eq_* = 0.4–0.44). As the strength level increased, the CCR increased with an increasing *C_eq_*. Moreover, for a constant strength level, the fillers of the R flux and F sheath produced larger CCRs than those of the B flux and S sheath. The large CCR was associated with a large amount of diffusible hydrogen in the WM (Figure 2b). This result is consistent with previous reports on cold cracking [26,27,28]. The CCR decreased as the content of the alloying constituents of the WM increased [26]. Hart [27] reported a method for controlling cold cracking in WMs, which depends on the diffusible hydrogen content. Diffusible hydrogen content below 5 mL/100 g significantly reduces cold cracking. In contrast, diffusible hydrogen contents exceeding 5 mL/100 g and a large fraction of hard phases increase the hardness; these have been reported as major factors affecting cold cracking [28]. Therefore, the CCR values of the WMs used in this study were determined to be associated with *C_eq_* and the diffusible hydrogen content.

**Figure 3:**
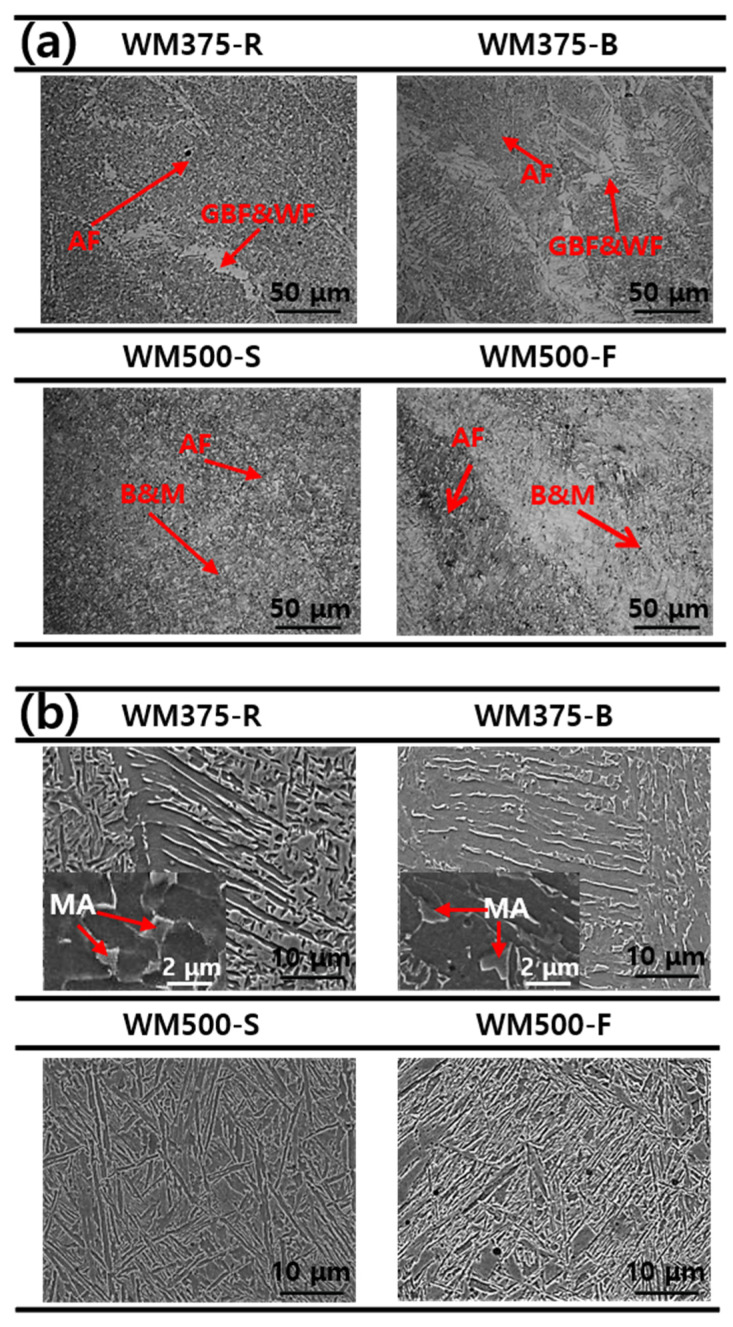
Microstructure of each WM measured using (**a**) LOM and (**b**) SEM.

Figure 3a,b present LOM and SEM photographs, respectively, of the representative microstructures of each WM. The WM375-R specimen contained GBF and WF in the AF matrix. Moreover, a small amount of MA phases was observed, as seen in Figure 3b. The WM375-B specimen exhibited a lower AF fraction in the dark area and a higher GBF fraction in the bright area than the WM375-R specimen. Specimens WM500-S and WM500-F exhibited primarily M and B, with no GBF and only a small amount of AF. With an increase in the strength grade of the WMs, the fractions of AF and GBF decreased, whereas those of M and B increased.

**Figure 4:**
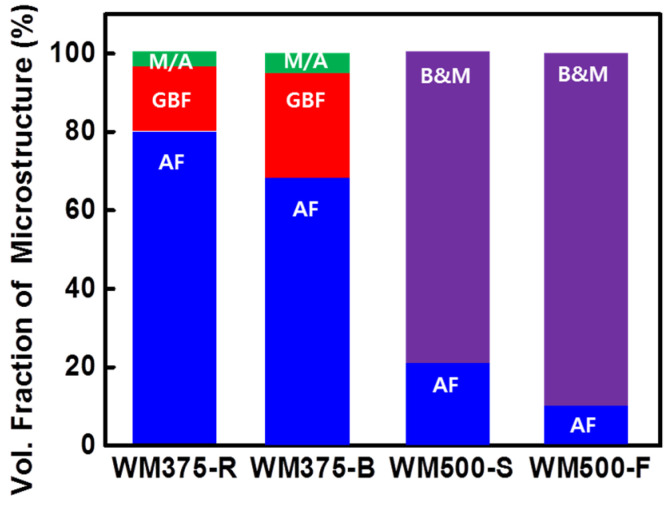
Volume fractions of various microstructures for the WMs.

Figure 4 shows the volume fractions of the quantitatively measured microstructures of the WMs. The 375 MPa grade WM had a microstructure comprising GBF, AF, and a small amount of MA, whereas M and B with AF were mainly present in the 500 MPa grade WM. With an increase in the strength grade of the WM, the major microstructural fraction changed from AF to M and B as *C_eq_* increased (0.30 < 0.31 < 0.40 < 0.44). The *C_eq_* values of WM375-B and WM375-R in the low-strength-grade WM were essentially the same. The fraction of GBF (which renders the WM vulnerable to cold cracking) in WM375-B was approximately 20% higher than that in WM375-R, and the fraction of AF in WM375-B was approximately 18% lower than that in WM375-R; however, the CCR of WM375-B was less than that of WM375-R. The low CCR of WM375-B was associated with the low content of diffusible hydrogen rather than the microstructural contribution.

Similarly, *C_eq_* was almost identical for WM500-S and WM500-F. Because the M/B fractions and diffusible hydrogen contents of WM500-F were approximately 10% and 1 mL/100 g higher than those of WM500-S, respectively, the CCR of WM500-F was also larger than that of WM500-S. Therefore, the M/B fraction and diffusible hydrogen content have a combined effect on the CCR of high-strength WMs. Despite an increase in the strength and *C_eq_*, WM375-R and WM500-F had approximately the same diffusible hydrogen contents, i.e., 3.64 and 2.39 mL/100 g, respectively. However, WM500-F exhibited a larger CCR (100%) than WM375-R (5%) did. This was due to the large amount of transformed low-temperature M/B microstructures for a high *C_eq_*. Moreover, the Ni content increased significantly with the strength grade of the WMs (Table 1). Specifically, Ni is a typical austenite-stabilizing element. Thus, the large Ni content stabilized the austenitic region and reduced the transformation temperature for γ ⇒ α, thereby reducing the fractions of GBF and fine AF and increasing those of M and B [29,30].

**Figure 5:**
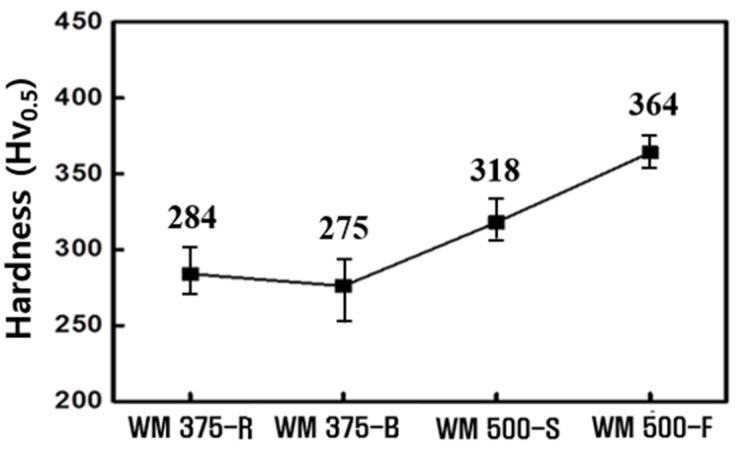
Average hardness of WMs for each strength grade.

Figure 5 shows the average hardness values of the WMs. The 375 MPa grade and 500 MPa grade WMs had hardness values of 275–284 Hv_0.5_ and 318–364 Hv_0.5_, respectively. The average hardness of the WMs increased with *C_eq_*. Specifically, WM500-F exhibited a larger amount of low-temperature transformed phases and a greater hardness than WM500-S did. Therefore, with an increasing fraction of the hard phase (Figure 4), the average hardness of the WM increased. The 500 MPa grade WMs had a microstructure consisting primarily of B and M (Figure 4). However, because the hardness was above 318 and 364 Hv_0.5_, M was likely the main component of the microstructure.

### 3.2. Effect of Groove Shape on Cold Cracking

**Figure 6:**
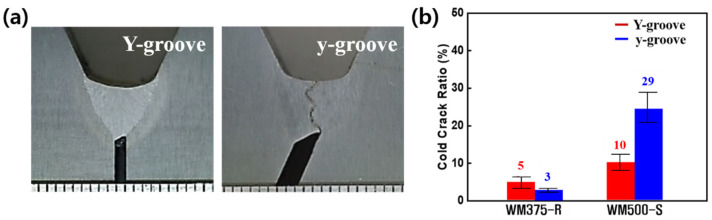
(**a**) Typical weld shape for WM500-S and (**b**) CCR for WM375-R and 500-S as a function of groove shape.

Figure 6a,b show the typical weld shapes and CCR of WM375-R and 500-S, respectively, as a function of the groove shape. Regardless of the groove shape, cold cracking occurred in all the WM specimens. The low-strength-grade WM exhibited similar, low CCRs for the Y- and y-grooves. However, the high-strength-grade specimens had a larger CCR, specifically for the y-groove compared with that for the Y-groove. Thus, the high-strength-grade WMs exhibited a significant difference in CCR with respect to the groove shape.

**Figure 7:**
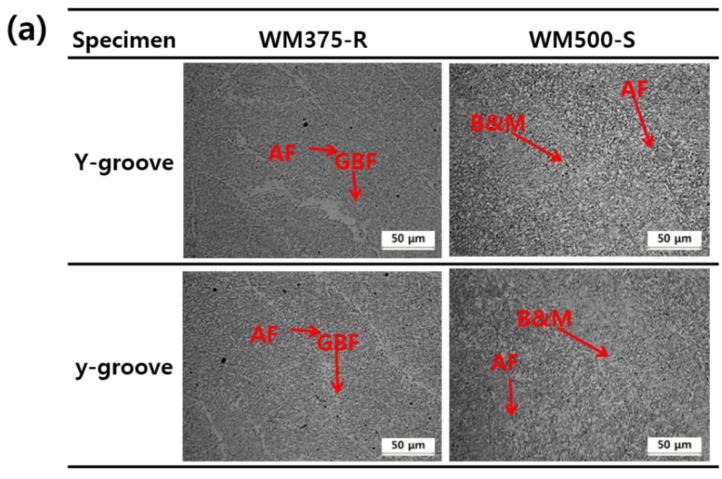

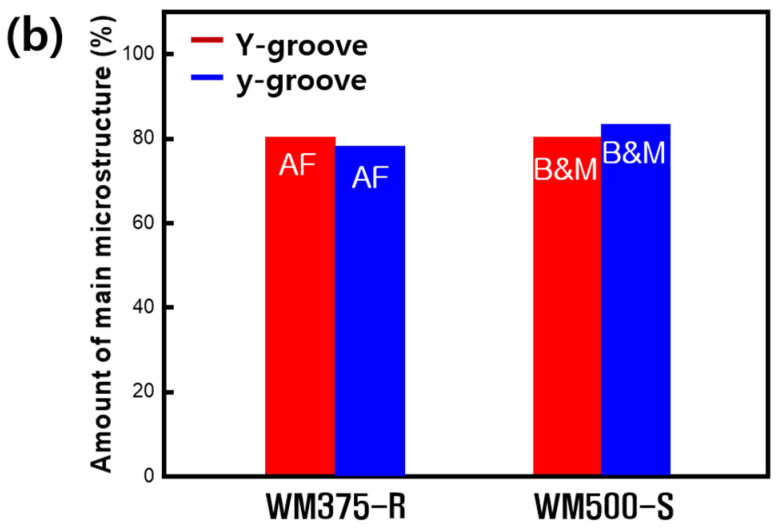
Microstructure of WM375-R and 500-S as a function of the groove shape: (**a**) light optical micrographs and (**b**) quantitative amount of main microstructural component.

Figure 7a,b show the LOM microstructure and the volume fraction of the main microstructure as a function of the strength grade and groove shape, respectively. WM375-R contained AF with some amount of GBF, while WM500-S contained B/M with a certain amount of AF. As shown in Figure 3, with an increasing strength grade, the main microstructure of the WMs changed from AF to B and M. The fraction of the main microstructure remained largely constant; however, this was regardless of the groove shape. Therefore, the CCRs of WM375-R and 500-S were not significantly dependent on the microstructure as a function of the groove shape.

**Figure 8:**
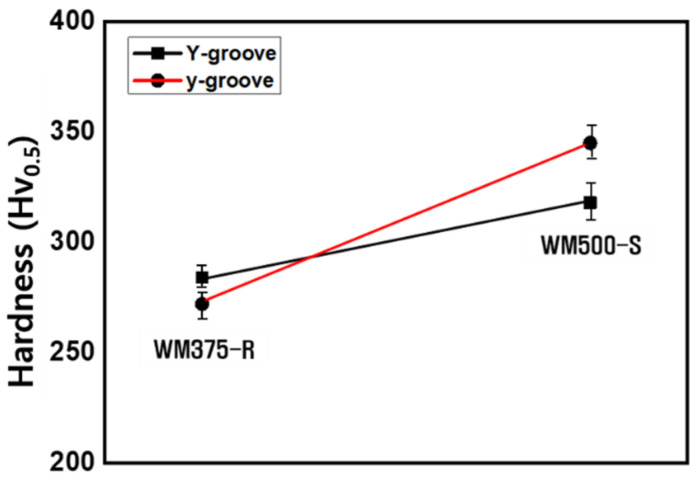
Average hardness of WM375-R and 500-S as a function of groove shape.

Figure 8 shows the average hardness values of WM375-R and 500-S as functions of the groove shape. As the strength grade increased, the average hardness increased because B and M became the main microstructure (Figure 7). The average hardness and main microstructure of the low-strength-grade WM were almost invariant with respect to the groove shape. However, the average hardness of the y-groove was slightly higher than that of the Y-groove in the high-strength-grade WM. This difference was independent of the microstructural behavior.

**Figure 9:**
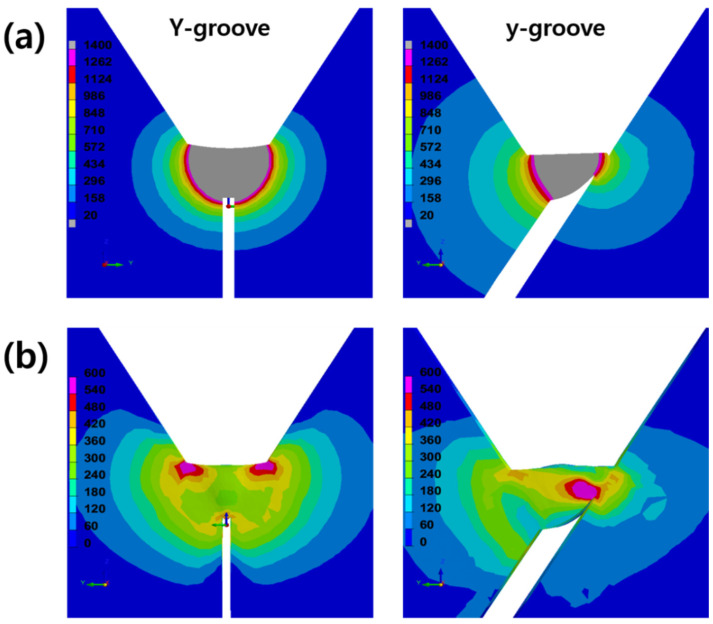
Simulation of heat distribution and stress concentration for 500 MPa grade WM as a function of groove shape: (**a**) weld bead geometry and (**b**) concentration of internal restraining stress.

Figure 9a,b show the weld bead geometry and residual stress concentration as a function of the groove shape, respectively, as determined via the SYSWELD analysis of WM500S. As shown in Figure 9a, the weld bead geometries and the degree of heat distribution varied significantly depending on the Y- and y-groove shapes. The weld bead of the Y-groove exhibited a symmetrical heat distribution. However, in the weld bead of the y-groove, an asymmetrical heat distribution was observed with respect to the groove shape. Therefore, the heat distributions on the left and right sides of the y-groove weld were significantly different. This difference in the heat distribution affected the internal restraining stress of each weld. The curvature radius of the sharp root notch in the y-groove was smaller than that in the Y-groove, and the stress concentration mainly occurred only on the sharp root notch, as shown in Figure 9b. For the y-groove weld, the location of cold-cracking initiation near the sharp root notch was identical (Figure 6b). However, the Y-groove weld exhibited a symmetrical stress concentration near the toe area of the weld surface. Specifically, the y-groove weld exhibited a larger area of stress concentration than the Y-groove weld. As indicated in Figure 6b, the 500 MPa grade WM showed a large CCR for the y-groove weld. In conclusion, the curvature radius of the notch plays an important role in determining the CCR for high-strength welds (500 MPa) but is not critical for determining the CCR for low-strength welds (375 MPa).

## 4. Conclusions

In this study, a comprehensive analysis of cold cracking was conducted, considering the alloying constituents, diffusible hydrogen content, and groove shape in WMs of different strength grades (YS 375 and 500 MPa). The following conclusions were drawn:

(1) The low-strength-grade specimens, WM375-R and WM375-B, had an almost identical *C_eq_* (0.3–0.31). WM375-B had a higher fraction of GBF and was more vulnerable to cold cracking than WM375-R. However, because WM375-R exhibited higher diffusible hydrogen content than WM375-B, the CCR of WM375-R was higher than that of WM375-B. The diffusible hydrogen content has a significant effect on cold cracking in low-strength WMs (YS 375 MPa).
(2) The high-strength-grade specimens, WM500-S and WM500-F, had almost the same *C_eq_* (0.4–0.44). Moreover, WM500-F had a greater hydrogen content and martensite/bainite fraction than WM500-S. Therefore, WM500-F exhibited a large CCR. The diffusible hydrogen content and the martensite/bainite fraction have a combined effect on the cold cracking of high-strength welds (YS 500 MPa).
(3) With an increase in the strength grade of the WM, the fractions of martensite (M) and bainite (B), which render the WM vulnerable to cold cracking, increase. The increased fractions of M and B increase the hardness and CCR of the WM.
(4) The low-strength WMs exhibited similar CCRs regardless of the Y/y-groove shape. However, for the high-strength WMs, the y-groove was simulated to have significant and unsymmetrical restraining stress exerted on the sharp root notch shape, therefore increasing the CCR experimentally. The Y/y-groove shape has a serious effect on cold cracking in high-strength WMs (YS 500 MPa).
(5) The current shipbuilding industry requires high-strength WMs. Therefore, for the welding of high-strength steel, it is necessary to select WMs with a low content of diffusible hydrogen and M and/or B to minimize cold cracking. Furthermore, the groove shape for CCR testing should be decided based on the morphology of real welds because the restraining stress, which depends on the groove shape, directly controls the occurrence of cold cracking.

## Figures and Tables

**Table 1:** Chemical compositions and diffusible hydrogen contents of various BMs and WMs. HD denotes the content of diffusible hydrogen.

Alloys	C	Si	Mn	Ni	Cr	Cu + Mo + B	*C_eq_*	*P_cm_*	HD (mL/100 g)
WM375-R	0.05	0.73	1.40	0.02	0.04	0.03	0.30	0.15	3.64
WM375-B	0.07	0.64	1.41	0.01	0.02	0.03	0.31	0.16	0.82
WM500-S	0.06	0.32	1.37	1.37	0.01	0.27	0.40	0.19	1.49
WM500-F	0.06	0.37	1.37	2.07	0.01	0.02	0.44	0.20	2.39
BM 235	0.16	0.28	0.68	0.01	0.02	0.01	0.28	0.20	-
BM 500	0.06	0.20	1.54	0.19	0.22	0.35	0.42	0.19	-

**Table 2:** Parameters used in SYSWELD simulation.

Model Parameters	Values
Material (base and weld metals)	Carbon steel
Ambient temperature (°C)	20
Element size (mm^3^)	~0.3
Heat source parameters (a_f_ + a_r_, b, c)	
a_f_ + a_r_	5 mm
b	5 mm
c	3 mm

## Data Availability

The data are available from the author upon request.
